# Moderating effect of social participation on the relationship between health status and depressive symptoms in older adults

**DOI:** 10.3389/fpubh.2025.1458961

**Published:** 2025-02-12

**Authors:** Yvonne Su Yong Ow, Chia-Shine Wei, Yang-Tzu Li

**Affiliations:** ^1^School of Pharmacy, College of Medicine, National Taiwan University, Taipei, Taiwan; ^2^Department of Long-Term Care, National Taipei University of Nursing and Health Sciences, Taipei, Taiwan

**Keywords:** depressive symptoms, disabilities, perceived health status, social participation, older adults

## Abstract

**Introduction:**

Depression is a common mental health problem among older adults and a leading cause of disability worldwide. Health status and social participation has been found significantly associated with depression in older adults. However, the role of social participation in the relationship between health status and depression remains unclear. This study aims to investigate the relationship between health status and depressive symptoms in older adults and the moderating effect of social participation in the relationship.

**Materials and methods:**

A cross-sectional study with data from the National Health Interview Survey (NHIS) in 2013. Older adults aged 65 years or older were included in the study. Multiple regression analysis was performed to examine the relationship between health status and depressive symptoms and assess the moderating effect of social participation.

**Results:**

Of the 2,731 participants, higher depressive symptoms were observed among older women, older adults who were single, living alone and having lower satisfaction on financial status. Regression analyses showed that activities of daily living (ADL) and instrumental activities of daily living (IADL) disability were positively associated with higher depressive symptoms. Perceived health status, cognitive functions and social participation were found negatively associated with depressive symptoms. A significant moderating effect of social participation between perceived health status and depressive symptoms was observed in this study.

**Discussion:**

The findings highlight the role of social participation in moderating the effect of perceived health status on depressive symptoms and suggest that promoting social participation among older adults may be helpful for reducing depressive symptoms.

## Background

Population aging is a global phenomenon. The older population in Taiwan has grown rapidly since 1993, driven by the declining fertility and increasing longevity. In 2020, 16% of the total population in Taiwan were aged 65 and over and it is expected to account for more than 20% by 2025, making Taiwan a super-aged society (1). The unprecedented growth in the older population results in an increased prevalence of non-communicable diseases, leading to a higher demand for care. Given the growing concern about the mental health of older adults, the long-term care nowadays focuses on the goal of maintaining functional abilities, addressing both physical and mental health problems in this population. Depression has become increasingly common among older adults in recent years, with a global prevalence ranged from 10% to 20% (2). Depression is one of the leading causes of disability and a significant contributor to the overall global burden of disease (3–5). Research has indicated that depression among older adults is associated with an increased risk of morbidity, mortality and suicide (6–8). Older adults suffering from depression usually require long-term or lifetime treatment that may affect their quality of life (9, 10).

Examining the risk and protective factors of depressive symptoms is important to prevent depression in older adults. Several studies have indicated an inverse relationship between functional status and depression in older adults (11–14). Studies have also found perceived health status, the indicator of overall health status, to be a significant predictor of depressive symptoms in older adults (15, 16), although the evidence remains limited. Older adults with poor perceived health have been shown to be significantly associated with a higher risk of depression (14, 17). Therefore, identifying the protective factors of depressive symptoms is important to protect against depression in older adults. Social participation is a key determinant of successful aging in the later stages of life. Previous research has suggested that social participation has beneficial effects on depressive symptoms in older adults. Older adults with higher social participation have been shown to have a lower risk of depressive symptoms (18, 19). However, the role of social participation in the relationship between health status and depressive symptoms of older adults remains unclear. Therefore, this study aims to evaluate the relationship between health status and depressive symptoms and explore the moderating effect of social participation on this relationship to bridge the knowledge gap. The findings from this study are important in preventing depression and promoting wellbeing in older adults.

## Methods

### Data source and study sample

This study used a secondary data obtained from the National Health Interview Survey (NHIS), which was conducted in 2013 through the joint efforts of the National Health Research Institutes, Taiwan Health Promotion Administration and National Bureau of Controlled Drugs. This study was approved by the Research Ethics Committee of National Taiwan University (No. 201911EM005) and written informed consent from participants was not required in accordance with national legislation and the institutional requirements. The NHIS was a cross-sectional sample survey with a nationally representative sample collected using a stratified multistage cluster sampling design. The information collected in the survey included individual health status, utilization of medical and preventive health services and health behavior. The NHIS covered the whole nation as the population and a probability proportional to size (PPS) method was used in the sample selection for the survey. Cluster analysis was conducted to divide 358 cities, townships or districts of Taiwan into seven strata based on proportional allocation. Two- or three-stage PPS was then used to gradually sample the cities, townships or districts, villages or neighborhoods and individual in each strata. In urban areas, the sample was selected through a two-stage PPS design with villages or neighborhoods selected as the primary sampling units (PSUs) at the first stage, followed by a selection of individuals in each PSU at the second stage. A total of 30,960 participants were surveyed between July 2013 and December 2013, with a complete response rate of 75.2% (23,296 respondents). The present study sample was collected from the survey questionnaires among participants aged 65 years or older and the data from other age groups were excluded in this study. A total of 2,731 participants were included for further analysis.

### Assessment instruments

#### Health status

Given that physical and mental health are equally important components of overall health in older adults, several measures were used to assess health status of the participants. Activities of daily living (ADL) and instrumental activities of daily living (IADL) were used to measure the physical disability; the Mini-Mental State Examination (MMSE) was used to measure the cognitive functions of the participants. In the ADL assessment, participants were asked to rate the levels of difficulty in doing six basic self-care tasks (feeding, bathing, dressing, toileting, transferring (in and out of bed) and walking) independently on a four-point Likert Scale, ranging from 0 (no difficulty) to 3 (unable to perform). The total scores ranged between 0 and 18, with higher scores indicating a greater level of disability. In the IADL assessment, participants were asked to rate the difficulty levels for eight living skills that are more complex than ADL, including food preparation, shopping, using a telephone, taking medications, doing housework, doing laundry, cleaning the house and managing money. The total scores of the 4-point Likert scale ranged from 0 to 24, with higher scores indicating a higher level of disability. Cognitive functions of the participants were measured using MMSE, which is widely used among older population. The participants were interviewed with a 30-point questionnaire to evaluate their cognitive status, with higher scores indicating a higher cognitive function. The participants were also asked to rate their perceived health status with a score ranging from 0 (very bad) to 4 (very good).

#### Depressive symptoms

The Center for Epidemiologic Studies Depression (CES-D) scale was used in the NHIS to measure depressive symptoms among the participants in the past week before being surveyed. The scale has 10 items rated on a 4-point Likert scale ranging from 0 (never or rarely less than a day) to 3 (always or 5–7 days). The CES-D, which is a reliable and valid measure of depression in older adults (20), measured both negative (8 items) and positive affects (2 items) among the participants and the scoring was reversed for the two positive affect items in the scale. Higher scores indicated a higher level of depressive symptoms.

#### Social participation

The literature has shown a lack of consensus on a standard definition and the domains of social participation. According to the Taiwan Active Aging Index (TAAI), important indicators for the evaluation of social participation include volunteering, grandparenting, political participation and community participation. Additionally, religious participation is an imperative aspect of life for many older adults. Research has shown an increase in religiosity with age, and older people tend to be more religious than younger generations (21, 22). Volunteering, grandparenting, community participation and religious participation were used as indicators for assessing social participation in the present study. Participants were asked to rate whether they were doing voluntary work and grandchild care on a 4-point Likert scale ranging from 0 (no) to 3 (often). Participants were asked to rate on a 3-point Likert scale ranging from 0 (no) to 2 (regular participation) about their community and religious participation. The scores obtained from the two different types of Likert scales were summed up, respectively, and multiplied by 4/3 to obtain two sub-scores. Total scores for the social participation of the participants were obtained by adding up the two sub-scores. Higher total scores indicated greater social participation. The median social participation score calculated in the study was used to categorize the study participants into low and high social participation groups for further analysis.

### Statistical analyses

Statistical analyses were performed using SPSS version 21, with a *p*-value of less than 0.05 considered statistically significant. Descriptive statistics were used to report the demographic characteristics of the study participants. Student's *t*-tests and one-way Analysis of Variance (ANOVA) were used to identify significant differences in depressive symptoms among participants with various demographic characteristics. Pearson's correlation was performed to assess the relationship between health status, social participation, and depressive symptoms. Multiple linear regression analysis was adopted in the study to investigate the associations of health status and social participation with depressive symptoms. In model A, potential confounders, including gender, living arrangement, marital status, perceived financial status, perceived health status, and social participation, were adjusted to account for their influence on depressive symptoms. Moderation analysis (model B) was then performed, where the interaction terms between social participation and health status were added in the model to examine whether social participation moderates the relationship between health status and depressive symptoms among older adults. A stratified analysis was subsequently performed to confirm the moderating effect of social participation. The association between health status with depressive symptoms was examined separately for participants categorized into low and high social participation groups.

## Results

Demographic characteristics and the difference in depressive symptoms among older adults in the present study are described in Table 1. There were significant differences in gender, living arrangement, marital status and perceived financial status with depressive symptoms in older adults. The results found that older women and single participants had significantly higher depressive symptoms than older men and married participants, respectively. In addition, older adults living alone and those who reported having lower perceived satisfaction with their financial status also showed significantly higher levels of depressive symptoms.

**Table 1:** Demographic characteristics and differences in depressive symptoms among older adults.

	**No of participants *n* (%)**	**CES-D scores (mean ±SD)**	***P*-value**
Gender			< 0.001
Male	1,298 (47.5)	3.717 ± 4.058	
Female	1,433 (52.5)	5.027 ± 4.963	
Living arrangement			< 0.001
Living alone	322 (11.80)	5.345 ± 4.959	
Living with spouse or family members	2,409 (88.20)	4.279 ± 4.538	
Marital status			< 0.001
Unmarried	979 (35.8)	5.363 ± 5.154	
Married	1,752 (64.2)	3.869 ± 4.168	
Perceived financial status			< 0.001
Very dissatisfied	111 (4.1)	9.045 ± 6.668	
Not satisfied	428 (15.7)	6.710 ± 5.650	
Neutral	1,278 (46.8)	4.275 ± 4.149	
Satisfied	805 (29.5)	3.014 ± 3.451	
Very satisfied	109 (4.0)	2.413 ± 3.110	
Total	2,731 (100)	4.404 ± 4.601	

Pearson's correlation analysis between health status, social participation and depressive symptoms in older adults is presented in Table 2. Depressive symptoms in older adults were found to be significantly correlated with their health status. A negative Pearson's correlation coefficient (*r* = −0.440, *p* < 0.001) was observed between perceived health status and depressive symptoms, indicating that higher perceived health status in older adults was significantly correlated with a lower level of depressive symptoms. Further, ADL and IADL disability were observed to have a significant and positive correlation (ADL: *r* = 0.290, *p* < 0.001; IADL: *r* = 0.364, *p* < 0.001) with depressive symptoms in older adults, whereas a higher score in cognitive function was found negatively correlated (*r* = −0.269, *p* < 0.001) with level of depressive symptoms in older adults. Moreover, social participation in older adults was also found to be negatively correlated with their depressive symptoms (*r* = −0.227, *p* < 0.001).

**Table 2:** Correlation coefficients between health status, social participation, and depressive symptoms.

**Factors**	**Perceived health status**	**ADL disability**	**IADL disability**	**MMSE scores**	**Social participation**	**CES-D scores**
Perceived health status	1					
ADL disability	−0.242^*^	1				
IADL disability	−0.328^*^	0.773^*^	1			
MMSE scores	0.231^*^	−0.275^*^	−0.384^*^	1		
Social participation	0.210^*^	−0.158^*^	−0.249^*^	0.202^*^	1	
CES-D scores	−0.440^*^	0.290^*^	0.364^*^	−0.269^*^	−0.227^*^	1

^*^p < 0.001.

The relationship of health status and social participation with depressive symptoms is shown in Table 3. The findings indicated that perceived health status (β = −1.453, *p* < 0.001), IADL disability (β = 0.163, *p* < 0.001), cognitive functions (β = −0.059, *p* = 0.014) and social participation (β = −0.198, *p* < 0.001) were significantly associated with depressive symptoms in older adults after adjusting for the potential confounders in the regression model. Specifically, better perceived health status, higher cognitive functions, and greater social participation are protective factors against depressive symptoms, while higher levels of IADL disability are identified as a risk factor. Notably, while ADL disability (β = 0.079, *p* = 0.116) was not statistically significant in model A, it became significant when interaction terms were included in model B (β = 0.179, *p* = 0.023).

**Table 3:** Multiple linear regression of health status and social participation on depressive symptoms.

	**CES-D scores**			
	**Model A**		**Model B**	
	β	***p*** **-value**	β	***p*** **-value**
Gender	0.735	< 0.001	0.709	< 0.001
Living arrangement	0.689	0.004	0.635	0.008
Marital status	−0.407	0.018	−0.438	0.011
Perceived financial status	−1.242	< 0.001	−1.237	< 0.001
Perceived health status	−1.453	< 0.001	−1.437	< 0.001
ADL disability	0.079	0.116	0.179	0.023
IADL disability	0.163	< 0.001	0.140	< 0.001
MMSE scores	−0.059	0.014	−0.064	0.008
Social participation	−0.198	< 0.001	−0.209	< 0.001
Social participation^*^perceived health status			0.269	0.001
Social participation^*^ADL disability			0.344	0.118
Social participation^*^IADL disability			−0.101	0.641
Social participation^*^MMSE scores			−0.059	0.437

Model A: Gender, living arrangement, marital status, perceived financial status, perceived health status and social participation were adjusted in the multiple linear regression model.

Model B: Interaction term between social participation and health status were included in the multiple linear regression model.

The moderation analysis in Model B further revealed that the interaction between social participation and perceived health status had a statistically significant effect on the depressive symptoms (β = 0.269, *p* = 0.001), indicating a significant moderating effect of social participation on the relationship between perceived health status and depressive symptoms among older adults. However, the interactions between social participation and other health-related factors, including ADL disability (β = 0.344, *p* = 0.118), IADL disability (β = −0.101, *p* = 0.641) and cognitive functions (β = −0.059, *p* = 0.437), were not statistically significant.

We further investigated and compared the effect of perceived health status on depressive symptoms in older adults categorized into low and high social participation groups (Table 4). The effect of perceived health status on depressive symptoms was greater among older adults in low social participation groups (β = −2.007, *p* < 0.001) than those in high social participation groups (β = −1.386, *p* < 0.001) after adjusting for gender, living arrangement, marital status and perceived financial status.

**Table 4:** Stratified analysis on depressive symptoms among participations in low and high social participation groups.

	**CES-D scores**			
	**Low social participation**		**High social participation**	
	β	***p*** **-value**	β	***p*** **-value**
Gender	0.786	< 0.001	1.005	< 0.001
Living arrangement	0.407	0.201	0.260	0.496
Marital status	−1.061	< 0.001	0.025	0.919
Perceived financial status	−1.406	< 0.001	−1.036	< 0.001
Perceived health status	−2.007	< 0.001	−1.386	< 0.001

## Discussion

Regarding the relationship between health, depression, and social participation in the literature, previous studies have explored the mediating role of functional health in the relationship between social participation and depression, the mediating effect of depressive symptoms between social participation and activity impairment, and the moderating effect of social participation on the relationship between depression and cognitive functioning in older adults (23–25). However, none have specifically examined the moderating role of social participation in the relationship between health status and depressive symptoms in older adults. This study is the first to investigate the moderating effect of social participation on the relationship between health status and depressive symptoms in this population. Different aspects of health status, including physical and mental impairments as well as perceived health status, were assessed in this study.

Our findings indicated that physical disability was positively correlated with depressive symptoms in older adults. Participants with higher levels of ADL and IADL disabilities were more likely to have depressive symptoms. This result is consistent with many recent studies that also found a positive association between physical disability and depression among older adults (11, 14, 26, 27). Similar results were also shown in other studies, in which older adults with higher ADL or IADL indicating greater physical ability. In this case, the association between physical ability and depression was reported in a negative direction. Lack of physical ability has been found to be associated with increased depressive symptoms among older adults (12, 13, 28, 29). Our regression model also revealed that IADL disability was positively associated with depressive symptoms. Interestingly, ADL disability was not significantly associated with depressive symptoms in the initial model but became significant when considering the interaction between social participation and health status in the regression model, suggesting that its relationship with depressive symptoms may be influenced by social participation. These complexities warrant further investigation to understand the nuanced role of ADL disability in the development of depressive symptoms in older adults, particularly its interactions with other factors such as social participation.

The current literature has shown that cognitive impairment often co-exists with late-life depression (30). A significant relationship between cognitive decline and depressive symptoms in older adults has been demonstrated in previous studies (14, 31–33). This finding is in accord with our study, which indicated a significant negative association between cognitive function and depressive symptoms in older adults. In contrast to the present study, a cross-sectional study reported that older adults with normal cognitive function were more prone to develop depressive symptoms. The possible reason is that older adults with normal cognitive function tend to be worried about their future life, which may cause an increased psychological burden and eventually lead to depression (19). Perceived health status is a subjective assessment of overall health status, including both physical and psychological health. Consistent with the existing literature (14–17, 28), this study confirmed that higher perceived health status in older adults was significantly associated with lower depressive symptoms. There is also evidence suggesting the reverse association and a bidirectional relationship between functional ability and depression in older adults, with depressive symptoms potentially contributing to functional impairments and vice versa (34, 35). Additionally, some longitudinal studies also highlight a complex dynamic in which functional ability and depression mutually influence one another over time (36, 37). These findings underscore the inconclusive nature of the evidence and the need for further research to disentangle these relationships.

Several studies have demonstrated that the lack of social participation is associated with a higher risk of depressive symptoms in older adults (18, 19). Higher social participation has been shown to have protective effects on depressive symptoms in late life, especially among older women (38, 39). This study also found an inverse relationship between social participation and depressive symptoms in older adults. The social participation investigated in our study included volunteering, grandparenting, and community and religious participation. Recent studies have indicated that engagement in voluntary work, community activities and leisure activities is associated with a reduced risk of depressive symptoms in older adults (40–43). Older adults with higher social participation have often been found to have better physical functioning, improved mental health and life satisfaction, decreased loneliness and a lower level of depression (44–46). Previous studies have also shown that grandparenting is associated with fewer depressive symptoms (47–49). Physical and mental health of older adults can be improved by engaging in grandparental care, which helps reduce loneliness and depression. Several studies have found improved psychological wellbeing among older adults who provide grandparental care to families (50, 51). Better self-rated health has also been reported among older women providing long-term non-residential grandchild care (52). Participation in religious activities has also found to be associated with a decreased risk of depression in older adults. Religiosity has been shown to protect against depression and help in depression recovery (53). The risk of depression has been found even lower among older adults who frequently attend religious services (54, 55).

Our study further investigated the effect of social participation on depressive symptoms and found a significant moderating role of social participation in the relationship between perceived health status and depressive symptoms among older adults. Specifically, older adults with high social participation experienced a weaker impact of perceived health status on depressive symptoms compared to those with low social participation. This finding aligns with Liu et al. (56), who reported that social support buffered the adverse effects of perceived health status on depression in older adults, suggesting a potential moderating role of social support in the connection between physical and mental health (56). Similarly, Mishra et al. (24) highlighted that higher social participation, combined with improved functional health were associated with a reduced risk of depression among older adults in India. However, their study identified functional health as a significant mediator in the relationship between social participation and depression (24). While Mishra et al. focused on mediation, our study provides complementary insights by demonstrating the moderating role of social participation in a similar context. Additionally, Ma et al. (23) investigated the moderating effect of social participation on the association between depression and cognitive functioning but found no significant interaction effects, while Yan et al. (25) demonstrated that social participation mitigated activity impairment in older adults with arthritis, partly by alleviating depressive symptoms (23, 25). These studies collectively underscore the multifaceted role of social participation in shaping mental and physical health outcomes among older adults. The non-significant moderating effects of social participation on the relationships between physical disability and depressive symptoms, as well as between cognitive function and depressive symptoms, observed in our study may stem from several factors. First, social participation might primarily influence subjective wellbeing (e.g., perceived health) rather than directly mitigating the challenges associated with physical or cognitive impairments. Second, the mechanisms linking physical and cognitive impairments to depression (e.g., pain or memory loss) may operate independently of social engagement, thereby limiting the moderating influence of social participation on these relationships.

While previous research has primarily focused on mediation effects or its influence on other outcomes, such as cognitive functioning or activity impairment, our study uniquely contributes by identifying the moderating effect of social participation on the relationship between perceived health status and depressive symptoms. However, several limitations warrant cautious interpretation of the results. First, the secondary data used in this study were obtained from the NHIS survey conducted in 2013. Given that the data are over a decade old, the results should be interpreted with caution, as they may not fully reflect current trends or societal shifts that have occurred since the data were collected. Additionally, self-reported rating scales were used to assess the participants' health status and depressive symptoms. The findings might differ if health status and depressive symptoms were clinically assessed. Second, the data were collected exclusively from older adults in Taiwan, which may limit the generalizability of the results to other populations or regions. Third, the cross-sectional design of this study restricts the ability to establish causal relationships between health status and depressive symptoms in older adults. The potential for reverse causation or a bidirectional relationship was not explicitly addressed in the present study. Fourth, our study analyzed older adults as a single group aged 65 years and older, which may mask important differences among age subgroups (e.g., 65–74, 75–84, and 85 and over). Future research should consider dividing the older population into smaller subgroups to provide more nuanced insights and avoid the assumption of homogeneity. Moreover, breaking down social participation into its specific components, such as volunteering, grandparenting, and community and religious participation, could reveal which types of engagement are most effective in protecting against depressive symptoms. Fifth, education level was not adjusted in our analysis due to data limitations, but it may be a potential confounder, as it could influence both health status and depressive symptoms. Future research could consider adjusting for education level to account for its possible confounding effect. Lastly, further longitudinal studies are also warranted to explore possible reasons for the insignificant moderating effect of social participation observed on the relationships between physical disability, cognitive function, and depressive symptoms among older adults in this study.

## Conclusion

Poor physical and cognitive health status as well as perceived health status were associated with increased level of depressive symptoms among older adults. This study also underscores the role of social participation in moderating the effect of the perceived health status on depressive symptoms in older adults. Promoting social participation among older adults, especially for those with poor perceived health status, may have the potential to reduce their symptoms of depression.

## Data Availability

The data analyzed in this study is subject to the following licenses/restrictions: The data that support the findings of this study are available from the Health and Welfare Data Science Centre (HWDSC) of the Ministry of Health and Welfare of Taiwan but restrictions apply to the availability of these data, which were used under license for the current study, and so are not publicly available. Data are however available from the HWDSC with permission of the Ministry of Health and Welfare of Taiwan. Requests to access these datasets should be directed to Health and Welfare Data Science Centre of the Ministry of Health and Welfare of Taiwan.
